# Addition of multi-locus variable number tandem repeat analysis to assist *Cryptosporidium parvum* foodborne outbreak investigation — France, 2025

**DOI:** 10.1016/j.fawpar.2026.e00343

**Published:** 2026-06-06

**Authors:** Fanny Chereau, Daphné Rageot, Lorraine Puzin, Frédéric Dalle, Genevieve L. Buser, Léa Lalan, Pierre-Emilien Dorgebray, Edith Laurent, Maria-Alexandra Stoica, Nathalie Fredriksen, Loic Favennec, Damien Costa, Henriette de Valk

**Affiliations:** a Santé Publique France, Infectious Diseases Division, 94415 Saint-Maurice, France; b Ministère de l'Agriculture, de l'Agro-alimentaire et de la Souveraineté Alimentaire – Direction Générale de l'Alimentation, Mission des urgences sanitaires, 75732 Paris, France; c Université Bourgogne Europe, CHU Dijon Bourgogne, National Reference Centre for Cryptosporidiosis, Microsporidia and other digestive protozoa, associate laboratory, 21070 Dijon, France; d National Reference Centre for Cryptosporidiosis, Microsporidia and Other Digestive Protozoa, Coordinating Centre, University hospital of Rouen, 76000 Rouen, Normandie, France

**Keywords:** *Cryptosporidium parvum*, Foodborne outbreak, Multi-locus variable number tandem repeat analysis (MLVA), Unpasteurized goat milk cheese, Lamb's lettuce, *Valerianella locusta*

## Abstract

*Cryptosporidium* spp. is a parasite that causes gastroenteritis in humans and is associated with waterborne and foodborne outbreaks. The international nomenclature for discrimination of *Cryptosporidium* isolates is based on sequence polymorphisms in the highly variable *gp60* gene. In 2025, the number of human cases of cryptosporidiosis caused by infection with *Cryptosporidium parvum gp60* genotype IIdA24G1 in France increased significantly and prompted public health authorities to investigate. During January through September 2025, 166 cases of infections with *C. parvum* genotype IIdA24G1 were identified, compared to less than 20 annual cases for previous years. To add further discrimination to *gp60* sequencing, multi-locus variable number tandem repeat analysis (MLVA) was performed by the National Reference Center and revealed two large distinct profiles. The national public health agency interviewed patients associated with each MLVA profile about water exposure and food consumption to identify sources of contamination. Food safety authorities obtained store loyalty card purchase information, performed food product traceback, and communicated with producers. Epidemiological investigations revealed high consumption of goat milk cheese for the first profile (17 of 23 cases interviewed) and pre-packaged lamb's lettuce for the second profile (19 of 21 cases interviewed). Product traceback identified food product origins, producers were notified and preventive measures were recommended. MLVA typing provided further granularity within a *C. parvum* IIdA24G1 genotype, which facilitated outbreak investigation and source identification. *Cryptosporidium-*specific monitoring and preventive hygienic measures should be considered for unpasteurized milk products and fresh produce.

## Highlights

• An unusual increase of *Cryptosporidium parvum gp60* genotype IIdA24G1 was identified.
• MLVA typing revealed two predominant profiles, representing 72% of cases.
• Two discrete MLVA profiles enabled targeted epidemiological investigations.
• Unpasteurized goat milk cheese and lamb's lettuce were suspected, respectively.

## 1 Introduction

*Cryptosporidium* spp. is recognized as a major cause of severe diarrhea and a common food-borne pathogen worldwide (Bouwknegt et al., 2018; GBD 2021 Diarrhoeal Diseases Collaborator, 2025; Innes et al., 2020). *Cryptosporidium* spp. is a parasite that infects humans and animals via fecal-oral transmission of oocysts from other human and zoonotic sources through a vehicle such as water, food, or the environment (Bouwknegt et al., 2018; Kyu et al., 2025). Gastrointestinal infection causes malabsorptive diarrhea, which may lead to dehydration and hospitalization, and chronic complications in the immunocompromised host.

Two species, *C. hominis* and *C. parvum,* account for most human cryptosporidiosis infections worldwide (Innes et al., 2020). *C. hominis* has a restricted host range and is primarily associated with anthroponotic transmission, whereas *C. parvum* has multiple reservoirs and is often linked to zoonotic transmission (Cacciò and Chalmers, 2016). In France, *C. parvum* represents between 63% and 90% of strains characterized during 2017–2024 (Costa et al., 2020, Centre National de Référence cryptosporidioses, 2025).

Due to its prolonged survival in moist environments, low infectious dose, and resistance to chlorine and other water treatment agents, *Cryptosporidium* spp. has been responsible for numerous waterborne outbreaks (Ryan et al., 2018; Zahedi and Ryan, 2020). Among the 12 French outbreaks with identified sources described in the literature (Beaudeau et al., 2008; Costa et al., 2022), 11 were linked to contaminated water exposure and one was of foodborne origin (Loury et al., 2019). The most frequently identified food sources in outbreaks involving *C. parvum* include fresh salads and herbs, raw vegetables, unpasteurized apple juice or cider, and products made from unpasteurized cow or goat milk (EFSA Panel on Biological Hazards et al., 2018; Ryan et al., 2018; Zahedi and Ryan, 2020).

In France, microbiological surveillance of *Cryptosporidium* spp. infections is coordinated by the National Reference Center (NRC) for Cryptosporidiosis, Microsporidiosis, and Other Digestive Protozoans, which receives samples from its network of public and private partner laboratories on a voluntary basis. The NRC performs species identification and *gp60* sequencing for *C. parvum* and *C. hominis* isolates*.* Since 2024, the NRC has implemented multi-locus variable number tandem repeat analysis (MLVA) for *C. parvum* isolates to improve strain differentiation. A MLVA profile is assigned according to international nomenclature (Robinson et al., 2022). While *gp60* genotyping relies on a single, highly polymorphic locus and is widely used for its simplicity and comparability, it can lack sufficient discriminatory power. In contrast, MLVA examines variations across multiple loci, providing higher resolution and allowing for a more precise distinction between isolates. In May 2025, the NRC notified Santé publique France, the national public health agency, of an increase in the number of *C. parvum* genotype IIdA24G1 infections observed since April 2025. The genotype IIdA24G1 is usually rare in France: < 20 isolates per year were identified by the NRC in 2022–2024, representing <3% of *C. parvum* isolates (NRC internal data). An outbreak investigation was launched to describe the unexpected increase of *C. parvum* genotype IIdA24G1 infections using discriminant MLVA typing, to determine the source or sources of infections using epidemiological interviews, and to recommend adapted control measures.

## 2 Materials and methods

### 2.1 Microbiological investigation

Human stool samples submitted to medical laboratories in the NRC network were analyzed for stool pathogens according to local protocols. *Cryptosporidium*-positive samples were forwarded to the NRC for further analysis, along with patient demographic information.

At the NRC, DNA was extracted from stool samples using the QIAamp PowerFecal Pro DNA kit (Qiagen, Courtaboeuf, Hilden, France) according to manufacturer's instructions. First, a real-time PCR was performed to confirm the presence of *Cryptosporidium* species (Hadfield et al., 2011). Next, *Gp60* subtypes were identified (Sannella et al., 2019), using a nested PCR using primers AL3531 (5′-ATAGTCTCCGCTGTATTC-3′)/AL3534 (5′- GCAGAGGAACC GCA TC-3′) used for C*.parvum/C. ho*min*is* discrimination (Alves et al., 2003) and followed by ATGFmod (5′- GAGATTGTCGCTCGTTATCG-3′) and GATR2 (5′- GATTGCAAAAACGGAAGG-3′) for *C. meleagridis* potential identification (Stensvold et al., 2014). Thermocycling conditions were: 94 °C for 3 min, followed by 40 cycles of 94 °C for 30 s (s), 50 °C for 60s and 72 °C for 60s and a final step at 72 °C for 7 min. Sequencing was performed using an AB3500 automated sequencer (Applied Biosystems, Illkirch, France). Analysis is based on the identification of a subtype family from DNA sequence variation downstream from a serine repeat, from which counting the composition of the repeats can separate subtypes within those families.

Since 2024, MLVA typing has been performed on *C. parvum* isolates, according to the seven-locus scheme of Robinson et al. (Robinson et al., 2022): cgd1_470_1429 (*cgd1*); cgd4_2350_796 (*cgd4*); cgd5_10_310 (*MSF*); cgd5_4490_2941 (*cgd5*); cgd6_4290_9811 (*cgd6*); cgd8_4440_NC_506 (*cgd8*); cgd8_4840_6355 (*MM19*). The amplification was divided into a four-plex PCR targeting *cgd1*, *cgd4*, *cgd8* and *MM19*, and a three-plex PCR targeting *MSF*, *cgd5* and *cgd6*. Both were launched at the same time on a T100 thermocycler (BIO-RAD, U.S.A.) with following PCR cycling conditions: initial *Taq* activation at +95 °C for 5 min, followed by 40 cycles of +95 °C for 30 s, +60 °C for 2 min, and + 72 °C for 30 s, and a final extension step at +60 °C for 30 min. PCR products were collected by short centrifugation and diluted 40-fold (four-plex PCR) or 50-fold (three-plex PCR) in HiDi Formamide (Thermofisher, U.S.A.). One μL of dilutions were then added to 10 μL of HiDi Formamide and 0.2 μL of GeneScan 600 Liz Size Standard 2.0 (Thermofisher, U.S.A.) inside a FrameStar 96-well PCR Plate (4titude, U.K.). Plates were loaded into the 3500 Series Genetic Analyser (Thermofisher, U.S.A.), and run settings were configured with the 3500 Data Collection Software using the Fragment Analysis application. Raw .fsa files were analyzed using the Genemapper software (version 4.1, Thermofisher, U.S.A.). All peak sizes were converted into number of repeats according to Robinson et al. (Robinson et al., 2022). The number of repeats were then assembled in the chromosomal order (*cgd1*, *cgd4*, *MSF*, *cgd5*, *cgd6*, *cgd8*, *MM19*) as a final result.

### 2.2 Epidemiologic investigation

Demographic data submitted with each specimen (age, sex, residential postal code, sampling date) were analyzed by Santé publique France for patterns in patient profile, timing, and location. A standardized questionnaire was used to interview cases and collect clinical data, including symptom onset and risk exposures to *Cryptosporidium* spp. in the 10 days preceding symptom onset. Environmental exposures (swimming, contact with pets or livestock) and food exposures (drinking water, raw milk and raw milk products, shellfish, unpeeled raw fruits and vegetables, unpasteurized cider or fruit juices) were investigated. Travel, participation in collective events (festivals, fairs, sports events), and details of restaurants visited during the exposure period were also recorded. Loyalty card numbers used for food purchases and consent for their use were obtained for traceability investigations.

### 2.3 Food traceability

Food traceability was conducted by French General Directorate for Food - Emergency Unit (Direction Générale de l'Alimentation, Mission des urgences sanitaires, DGAl-MUS) and local food safety authorities. When a food item was strongly suspected due to patients' interview data, the food safety authorities used store loyalty cards to examine the purchase history for a one-month period prior to the onset of symptoms. If purchase of the suspected food item was documented, traceability details were collected – brand, lot numbers sold on the purchase date in question, health mark – in order to identify the distributer and producer. When cases reported eating outside of the home, local food safety authorities contacted the restaurant or caterer about foods served on the date in question and any traceability information available.

### 2.4 Ethics

This investigation fell within the framework of the CNIL (French National Commission for Informatics and Freedoms) authorization for urgent investigations (n°341194v42).

## 3 Results

**Fig. 1:**
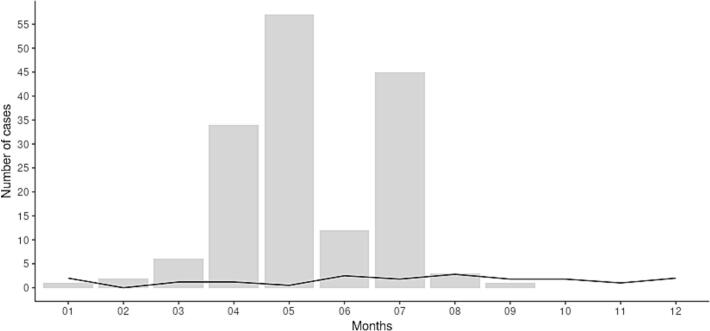
Monthly count of IIdA24G1 *Cryptosporidium parvum* isolates over January to September 2025 (*n* = 166, bars) compared to average in 2020–2024 (*n* = 75, line), France.

Between 1 January and 30 September 2025, the NRC identified 166 *C. parvum* strains belonging to the IIdA24G1 genotype, compared to 75 for the entire 5-year period between 2020 and 2024 (Fig. 1). Most cases of infection (*n* = 148) were identified between April and July 2025.

**Fig. 2:**
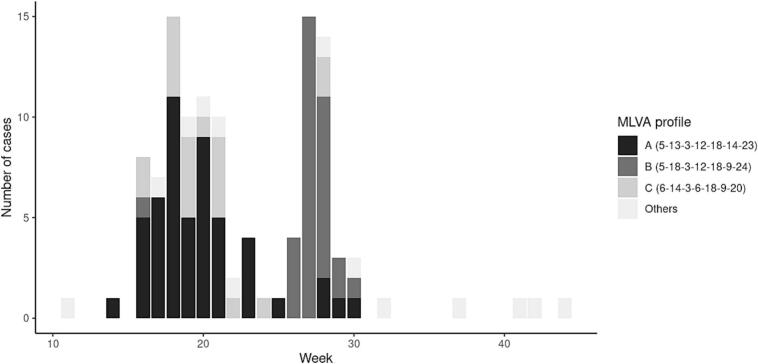
Weekly count of IIdA24G1 *Cryptosporidium parvum* isolates over January to September 2025 by MLVA profile (*n* = 115), France.

In total, 115 of the 166 *C. parvum* strains were successfully typed by MLVA. Sixteen different profiles were identified: the three most frequent profiles were (A) 5-13-3-12-18-14-23 (51 isolates, 44%); (B) 5-18-3-12-18-9-24 (32 isolates, 28%); and (C) 6-14-3-6-18-9-20 (19 isolates, 17%), followed by less frequent profiles (1 to 2 isolates per profile) (Fig. 2).

The two most frequent MLVA profiles, profiles A and B, were investigated as outbreaks. We further defined an outbreak period for each profile: 14 April to 08 June 2025 (week 16 to week 23) for profile A; and 23 June to 27 July 2025 (week 26 to week 30) for profile B.

### 3.1 Outbreak investigation of profile A (MLVA 5-13-3-12-18-14-23)

A total of 45 outbreak cases with profile A occurred between 14 April and 08 June 2025. The median age of the cases was 29 years (range: 10–75); 51% were women. Cases resided across eight regions of France.

Santé publique France interviewed 23 cases. Onset dates ranged from 26 March to 11 May 2025. The median duration of symptoms was 10 days (range: 5–42). No cases were hospitalized nor died. Among them, 17 (74%) reported consumption of goat cheese. Three cases mentioned the same variety of soft-ripened raw goat milk cheese. Three cases reported consuming goat milk cheese outside home: in restaurants (2 cases) or in a collective catered setting (1 case). No other food consumption was common to more than 50% of cases.

Review of loyalty card purchases confirmed purchase of the same raw goat milk cheese variety in 5 cases; all 5 products traced back to the same cheese producer. The DGAl-MUS confirmed that the suspected raw goat milk cheese variety was served in the two restaurants mentioned by cases, though further traceability details could not be obtained. The local food safety authorities notified the cheese producer of the human cases of cryptosporidiosis reporting consumption of their raw goat milk cheese product.

Traceability of the cheese identified two dairy farms as the source of the milk used to produce the purchased batches. The milk suppliers reported no episodes of cryptosporidiosis within their herds. The cheese batches identified following the traceability investigations had expired use-by dates (7 May 2025 and 23 May 2025) at the time of the investigation. Therefore, no remaining cheese or milk product was available for testing.

### 3.2 Outbreak investigation of profile B (MLVA 5-18-3-12-18-9-24)

Thirty-one outbreak cases with profile B were identified between 24 June and 18 July 2025. The median age of the cases was 28 years (range: 9–45); 67% were women. Cases resided across 6 regions of France.

Santé publique France interviewed 21 cases. Onset dates ranged from 14 June to 07 July 2025. The median duration of symptoms was 14 days (range: 7–21). No cases died and one case was hospitalized. Nineteen (90%) cases reported consumption of pre-packaged, bagged lamb's lettuce (*Valerianella locusta*) from diverse brands and places of purchase. No other food consumption was common to more than 50% of cases.

Review of loyalty card purchase confirmed purchase of pre-packaged lamb's lettuce in 6 cases; 5 of the 6 originated from the same distributer. Food safety authorities informed the lamb's lettuce distributer and its producers of the outbreak and that lamb's lettuce was the suspected vehicle. Local food safety authorities recommended review of good agricultural practices, including water quality from seed to harvest to washing; prevention of animal waste contamination; and inclusion of *Cryptosporidium* oocysts contamination in production risk assessments. Due to the perishability of the fresh product, no samples from the period of suspected contamination could be tested.

## 4 Discussion

Between April and July 2025, in France, we observed an increase of human infections of cryptosporidiosis due to *Cryptosporidium parvum* genotype IIdA24G1. MLVA typing enabled discrimination of several genetic clusters within the overall increase of the IIdA24G1 genotype. Traditional epidemiological approaches were then used to identify the probable, distinct sources of infection for two predominant MLVA clusters. This aligns with previous findings that MLVA typing is more discriminatory than *gp60* genotyping alone for detecting clustered cases, thereby aiding the epidemiologic investigation (Robinson et al., 2022).

The MLVA profile A was associated with consumption of raw goat milk cheese, while MLVA profile B was associated with consumption of pre-packaged lamb's lettuce. While traceback identified a producer at the source of each product, no sampling of food products was conducted. At the time the suspected products were identified, no further cases had been recently reported, and the suspected batches were no longer on the market. Therefore, sampling of agricultural water, production environments, primary products (milk, unharvested lamb's lettuce) or animals' feces was not necessary for outbreak management purposes. Both outbreaks resolved spontaneously without implementation of specific control measures, suggesting isolated source contamination events.

These outbreaks are the first documented outbreaks in France involving the IIdA24G1 genotype. In the literature, outbreaks and clusters involving the IIdA24G1 genotype have identified consumption of fresh vegetables, such as pre-packaged salads, as a risk factor for infection (Gherasim et al., 2012; Ryan et al., 2018). Although IId subtype is associated with livestock reservoirs in Europe (Zahedi and Ryan, 2020), the IIdA24G1 genotype infections had not previously been linked to unpasteurized milk products.

Nevertheless, the risk of cryptosporidiosis associated with the consumption of raw milk and raw milk products is well-documented (Bulumulla et al., 2025). In France in 2017, a strain of hyper-transmissible *C. parvum* genotype IIaA15G2R1 caused gastroenteritis in 180 students after they consumed an unpasteurized cow milk product (*fromage blanc*) served by the school cafeteria, the same genotype was isolated from the feces of calves housed near the cheese production room on the farm (Loury et al., 2019). Goat milk consumption can also be a source of cryptosporidiosis: in 2014, US public health authorities published an outbreak of *C. parvum* genotype IIaA16G3R1 among 11 cases associated to unpasteurized goat milk consumption (Rosenthal et al., 2015). Further exploration into the prevalence of *C. parvum* in goat farms, in raw milk products and throughout the farm-to-consumer chain is warranted. *C. parvum* is known to circulate in goat herds (Delafosse et al., 2006) and is responsible for diarrheal episodes in young animals in France and other European countries (Díaz et al., 2018). Assessing environmental contamination, particularly in water sources, could provide insights into how the milk becomes contaminated.

Pre-packaged salads have been implicated in multiple documented cryptosporidiosis outbreaks since 2008 (Åberg et al., 2015; Insulander et al., 2008; McKerr et al., 2015; Pönka et al., 2009; Ryan et al., 2018), and *C. parvum* DNA has been detected on fresh produce (Caradonna et al., 2017; Dixon et al., 2013; Suleiman et al., 2024). While the specific events leading to contamination of fresh produce are rarely identified, untreated agricultural irrigation water is considered the most probable source of Cryptosporidium species oocysts (Dixon et al., 2013; Golomazou et al., 2024). The potential for lamb's lettuce to become contaminated during the germination stage and carry viable *Cryptosporidium* oocysts into mature plants at harvest has been demonstrated (Kubina et al., 2023). Washing with or without chlorinated water, rinsing or spinning decreased slightly, but did not remove, infective oocysts.

In Europe, there is no legal tolerance threshold for *Cryptosporidium* spp. oocysts in food matrices and no specific regulation governing its detection. According to the European guidance on addressing microbiological risks in fresh produce at primary production through good hygiene (2017/C 163/01) and the European guide on good hygiene practices in the production of artisanal cheese and dairy products (European Commission, 2016), *Cryptosporidium* is not identified as a risk requiring specific consideration in operators' HACCP (Hazard Analysis and critical Control Points) procedures in routine situations. However, general hygiene best practices, particularly those related to water quality and fecal contamination risk, still apply. In future outbreaks of cryptosporidiosis, it could be informative to perform exploratory environmental (irrigation water, soil), animal (herd feces samples), and product sampling, in order to expand our knowledge of the circulation and prevalence of this parasite at different steps along unpasteurized and fresh food production.

Current human cryptosporidiosis surveillance in France does not ensure exhaustive case finding and complete geographical representation*.* Human cryptosporidiosis is not subject to mandatory reporting and surveillance relies on a voluntary reporting of cases by a national network of medical laboratories. However, this limitation does not preclude the accurate identification and investigation of outbreaks of a significant magnitude.

## 5 Conclusion

We report these two foodborne outbreaks due to *Cryptosporidium parvum* to increase visibility of such events to professionals at all levels of the food chain: fresh produce and dairy producers, food safety authorities, clinicians, laboratories and public health authorities. The added specificity of MLVA to differentiate clusters within the same genotype was valuable to enable targeted epidemiological investigation to identify the likely sources of infection. Opportunities for prevention include raising awareness and promoting good agricultural and hygiene practices at food production sites. A monitoring plan, conducted by the French General Directorate for Food, to assess the distribution of *C. parvum* oocysts in unpasteurized dairy products and fresh produce, could expand our knowledge of *C. parvum* contamination in high-risk food products in France.

## CRediT authorship contribution statement

**Fanny Chereau:** Writing – original draft, Visualization, Supervision, Investigation, Formal analysis, Data curation, Conceptualization. **Daphné Rageot:** Writing – original draft, Visualization, Investigation. **Lorraine Puzin:** Writing – review & editing, Investigation. **Frédéric Dalle:** Writing – review & editing, Investigation. **Genevieve L. Buser:** Writing – review & editing, Supervision, Investigation. **Léa Lalan:** Investigation. **Pierre-Emilien Dorgebray:** Investigation. **Edith Laurent:** Investigation. **Maria-Alexandra Stoica:** Investigation. **Nathalie Fredriksen:** Writing – review & editing, Project administration, Investigation. **Loic Favennec:** Writing – review & editing, Project administration. **Damien Costa:** Writing – review & editing, Supervision, Project administration, Investigation. **Henriette de Valk:** Writing – review & editing, Project administration.

## Declaration of competing interest

The authors have declared no conflict of interest regarding the original manuscript entitled *“*Addition of multi-locus variable number tandem repeat analysis to assist *Cryptosporidium parvum* foodborne outbreak investigation — France, 2025*.”*
